# Activation of TREK-1, but Not TREK-2, Channel by Mood Stabilizers

**DOI:** 10.3390/ijms18112460

**Published:** 2017-11-19

**Authors:** Eun-Jin Kim, Dong Kun Lee, Seong-Geun Hong, Jaehee Han, Dawon Kang

**Affiliations:** Department of Physiology, College of Medicine and Institute of Health Sciences, Gyeongsang National University, Jinju 52727, Korea; eunjin1981@hanmail.net (E.-J.K.); dklee@gnu.ac.kr (D.K.L.); hong149@gnu.ac.kr (S.-G.H.); jheehan@gnu.ac.kr (J.H.)

**Keywords:** bipolar disorders, depression, mood stabilizers, tandem pore domain potassium channels

## Abstract

Earlier studies have demonstrated that the tandem pore domain weak inward rectifying K^+^ channel (TWIK)-related K^+^ (TREK)-1 channel is inhibited by antidepressants and is associated with major depression. However, little is known about the effect of mood stabilizers that are commonly used for treatment of bipolar disorder on TREK channels, members of the two-pore domain K^+^ (K_2P_) channel family. This study sought to investigate the effect of mood stabilizers on TREK-1 and TREK-2 channels. HEK-293A cells were transfected with human TREK-1 or TREK-2 DNA. The effect of mood stabilizers on TREK-1 and TREK-2 was studied using the patch clamp technique. Changes in TREK protein expression by mood stabilizers were studied in the HT-22 mouse hippocampal neuronal cells using western blot analysis. Lithium chloride (LiCl, 1 mM), gabapentin (100 μM), valproate (100 μM), and carbamazepine (100 μM) increased TREK-1 currents by 31 ± 14%, 25 ± 11%, 28 ± 12%, and 72 ± 12%, respectively, whereas they had no effect on TREK-2 channel activity. In addition, western blot analysis showed LiCl and carbamazepine slightly upregulated TREK-1 expression, but not TREK-2 in the HT-22 cells. These results suggest that TREK-1 could be a potential therapeutic target for treatment of bipolar disorders as well as depression, while TREK-2 is a target well suited for treatment of major depression.

## 1. Introduction

TWIK-related K^+^ (TREK)-1 and TREK-2 channels, members of the two-pore domain K^+^ (K_2P_) channel family, are inhibited by antidepressants (fluoxetine, norfluoxetine, and paroxetine) and antipsychotics (fluphenazine, chlorpromazine, haloperidol, flupenthixol, loxapine, and pimozide) in a concentration-dependent and reversible manner [1,2,3,4]. Mice with deletion of TREK-1 were more resistance to developing depressive behavior, as judged by forced swim test, tail suspension test, conditioned suppression of motility, learned helplessness, and novelty suppressed feeding and displayed a doubled 5-hydroxytryptamine (5-HT) neuronal activity compared to wild type mice [3]. Therefore, the TREK-1 channel has been presented as a new potential therapeutic target for the treatment of depression, as it is the first ion channel implicated in the pathophysiology of depression [3,5,6]. TREK-1 and TREK-2 share similar biophysical and pharmacological properties in a variety of cells [7]. However, relatively little attention has been focused on TREK-2. At this point, we sought to ask whether both TREK-1 and TREK-2 channels (TREKs) could be involved in mania as well as depression. 

Bipolar disorder, also referred to a manic-depressive illness, is distinguished by a cycling between depressive episodes and mania that causes atypical moves in a person’s mood, energy, and ability to work. Mania is diagnosed by periods of excitement or irritability and hyperactivity, increased talkativeness, flight of ideas, inflated self-esteem, decreased need for sleep, distractibility, attack, and imprudent behavior with inadequate discernment [5,8]. Most of the signs that classify a manic episode cannot be replicated in preclinical research [5], thus research on mania is limited.

Mood stabilizers are commonly used for treatment of bipolar disorder. Therefore, we applied mood stabilizers on cells transfected with TREKs in an attempt to identify the relationships between current bipolar disorder treatments and TREKs. Until now, there have been no reports regarding the relationship between TREK channels and mood stabilizers. Here, we investigate the effect of mood stabilizers, such as lithium chloride (LiCl), valproate, and carbamazepine on TREK channel activity and expression to determine if TREK channels could serve as targets of mood stabilizers. 

## 2. Results

### 2.1. Effect of Antidepressants and Antipsychotics on TREK-1 and TREK-2 Current

**Figure 1:**
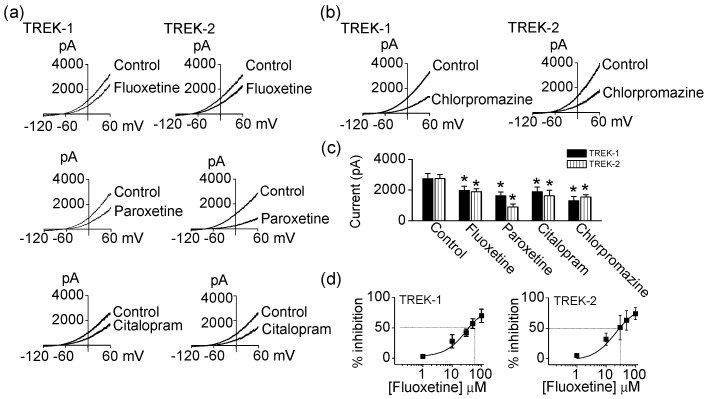
Effect of antidepressants and antipsychotics on TWIK-related K^+^ (TREK) channels. (**a**) Inhibition of TREK currents by fluoxetine, paroxetine, and citalopram. Whole-cell currents were recorded from HEK-293A cells expressing TREK-1 and TREK-2 before and after application of antidepressants. Cell membrane potential was held at −80 mV, and ramp pulses were applied from −120 mV to +60 mV. Pipette solution contained 150 mM KCl and bath solution contained 5 mM KCl and 135 mM NaCl; (**b**) Inhibition of TREK currents by chlorpromazine. Same protocol for recording of whole-cell currents as in a; (**c**) Summary of effect of fluoxetine (TREK-1, *n* = 10; TREK-2, *n* = 6), paroxetine (TREK-1, *n* = 10; TREK-2, *n* = 7), citalopram (TREK-1, *n* = 10; TREK-2, *n* = 7), and chlorpromazine (TREK-1, *n* = 4; TREK-2, *n* = 16) on TREK currents. Each bar is the mean ± standard deviation (SD ) of five experiments. Asterisk (*) indicates a significant difference against the control without application of fluoxetine, paroxetine, citalopram, or chlorpromazine (*p* < 0.05); (**d**) Dose-dependent effect of fluoxetine on TREK-1 (*n* = 8) and TREK-2 (*n* = 4) current. The inhibition of TREK current by increasing concentrations of fluoxetine (1 to 100 μM).

To revisit and analyze the effect of antidepressants and antipsychotics on TREKs in more detail, fluoxetine, paroxetine, citalopram, and chlorpromazine (an antipsychotic) were applied to bath solution containing cells transfected with human TREK-1 or human TREK-2. Application of 10 μM fluoxetine, 20 μM paroxetine, 100 μM citalopram, and 30 μM chlorpromazine significantly inhibited TREK-1 currents by 28 ± 11%, 40 ± 9%, 31 ± 10%, and 52 ± 10%, respectively (*p* < 0.05, Figure 1a–c). TREK-2 current was also decreased by fluoxetine (10 μM), paroxetine (20 μM), citalopram (100 μM), and chlorpromazine (100 μM) to 68 ± 10%, 33 ± 7%, 59 ± 6%, and 57 ± 5%, respectively (*p* < 0.05, Figure 1a–c). The inhibition was dose-dependent and reversible. Fluoxetine-induced inhibition is represented in Figure 1d. As shown in Figure 1d, the half maximal inhibitory concentration (IC_50_) values for the inhibition of TREK-1 and TREK-2 currents by fluoxetine were within 40 μM (37.9 ± 7.7 μM for TREK-1, 28.7 ± 7.6 μM for TREK-2).

### 2.2. Effect of Mood Stabilizers on TREK-1 and TREK-2 Current 

**Figure 2:**
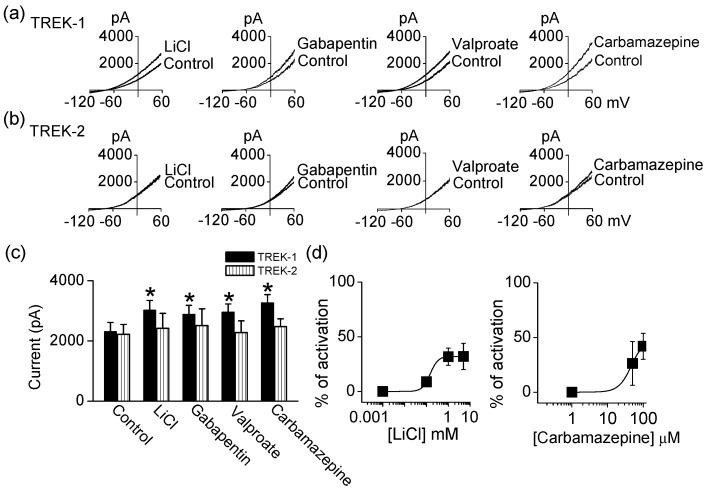
Comparison of effect of mood stabilizers on TREK-1 and TREK-2 current. (**a**) Effect of LiCl, gabapentin, valproate, and carbamazepine on TREK-1; (**b**) No effect of LiCl, gabapentin, valproate, and carbamazepine on TREK-2 activity; (**c**) Summary of effect of LiCl, gabapentin, valproate, and carbamazepine on TREK currents. Each bar is the mean ± SD of five experiments. Asterisk (*) indicates a significant difference against the control without application of drugs (*p* < 0.05); (**d**) Dose-dependent effect of LiCl and carbamazepine on TREK-1 channel. The activation of TREK-1 current by increasing concentrations of LiCl (0.001 to 1 mM) and carbamazepine (1 to 100 μM).

In agreement with previous studies, antidepressants and antipsychotics inhibited the activity of TREKs. To investigate whether mood stabilizers modulate TREKs, LiCl, gabapentin, valproate, and carbamazepine were applied to bath solution. Application of LiCl (1 mM), gabapentin (100 μM), valproate (100 μM), and carbamazepine (100 μM) significantly activated TREK-1 by 31 ± 14%, 25 ± 11%, 28 ± 12%, and 42 ± 12%, respectively (*n* = 5, *p* < 0.05, Figure 2a,c). However, these drugs failed to activate TREK-2 currents (Figure 2b,c). The activation of TREK-1 by LiCl, gabapentin, valproate, and carbamazepine was reversible and dose-dependent. As shown in Figure 2d, carbamazepine activated TREK-1 current in a dose-dependent manner. LiCl concentrations over 1 mM had no additional effect on TREK-1 activation. TREK-1 is more sensitive to carbamazepine than other drugs. 

**Figure 3:**
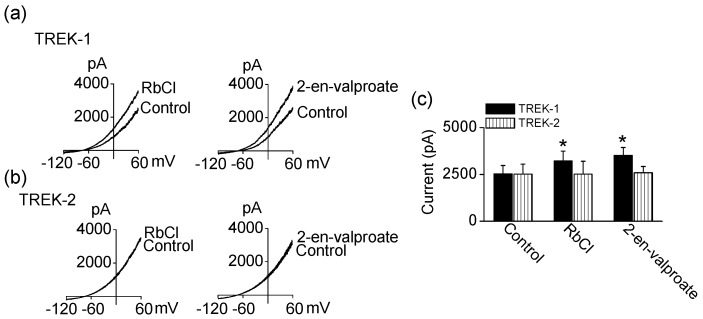
Effect of compounds structurally similar to LiCl and valproate on TREK-1 and TREK-2 current. (**a**) Effect of RbCl and 2-en-valproate on TREK-1; (**b**) No effect of RbCl and 2-en-valproate on TREK-2 activity; (**c**) Summary of effect of RbCl and 2-en-valproate on TREK currents. Each bar is the mean ± SD of five experiments. Asterisk (*) indicates a significant difference against the control without application of drugs (*p* < 0.05).

TREK-1 and TREK-2 channel activities were further tested using compounds structurally similar to LiCl and valproate. RbCl and 2-en-valproate (a metabolite of valproate) produced similar effects on TREK-1 and TREK-2 channel activities as LiCl and valproate. Application of RbCl (1 mM) and 2-en-valproate (100 μM) significantly activated TREK-1 by 27 ± 13% and 39 ± 17%, respectively (Figure 3a,c). Like LiCl and valproate, they did not affect TREK-2 currents (Figure 3b,c). These mood stabilizers also increased TREK-1 currents when recording in both inside-out and outside-out patch configurations. 

### 2.3. Alteration in TREK Protein Expression by Antidepressants and Mood Stabilizers in Hippocampal Neuronal Cell Line

**Figure 4:**
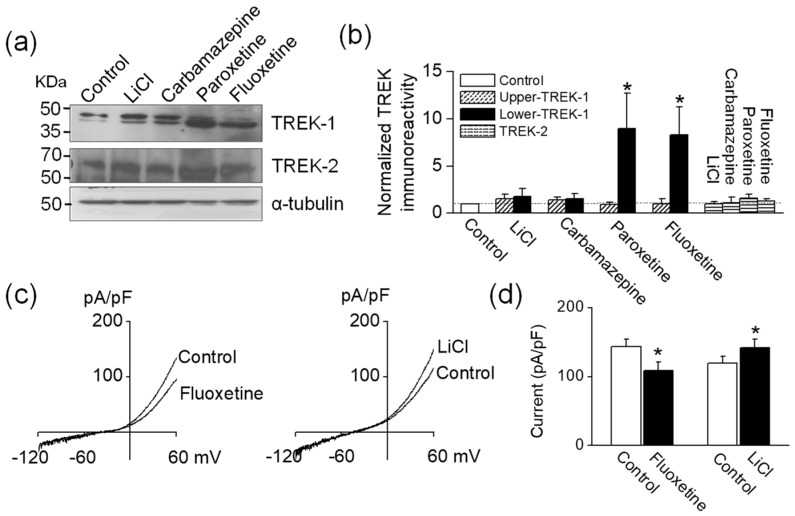
Effect of antidepressants and mood stabilizers on TREK channels expressing in HT-22 cells. (**a**) Western blot analysis of TREK-1 and TREK-2 in HT-22 cells treated with antidepressants and mood stabilizers. Molecular weight is indicated on the left side of the blot. In competition experiments pre-absorbed with a two-fold excess of the antigenic peptide, no signals were observed. (**b**) The bar graph shows normalized protein levels of TREK-1 and TREK-2 in HT-22 cells treated by LiCl, carbamazepine, paroxetine, and fluoxetine. The expression levels were normalized to α-tubulin. Each bar represents the mean ± SD of six experiments. (**c**) Whole-cell current tracings show the effect of fluoxetine and LiCl in HT-22 cells. (**d**) Each bar represents the mean ± SD of 13 patches. Asterisk (*) indicates a significant difference against the control without application of drugs (*p* < 0.05).

To identify whether TREK protein expression is changed by antidepressants and mood stabilizers, mouse hippocampal neuronal cell line HT-22 cells were treated with LiCl, carbamazepine, paroxetine, or fluoxetine. Of mood stabilizers, LiCl and carbamazepine were selected because LiCl is the most commonly used drug for the treatment of bipolar disorder, and carbamazepine is the most effective modulator of TREK-1. Using anti-TREK-1 antibodies purchased from two different companies, we detected two bands of ~50 kDa in HT-22 cells. The expression levels of TREK proteins were normalized to α-tubulin. The intensities of both bands showing the levels of TREK-1 expression were slightly increased by LiCl or carbamazepine (LiCl intensity: 1.4 ± 0.2 (upper) versus 1.8 ± 0.4 (lower) and carbamazepine intensity: 1.4 ± 0.3 (upper) versus 1.5 ± 0.4 (lower), Figure 4a). The intensities were normalized to the corresponding control. In contrast, HT-22 cells treated with paroxetine and fluoxetine showed different pattern between the upper band and the lower band. The upper band was downregulated (paroxetine: 0.8 ± 0.3 and fluoxetine: 0.6 ± 0.3), whereas the lower band was significantly upregulated (paroxetine: 9.0 ± 3.7 and fluoxetine: 8.3 ± 3.0, *n* = 6, *p* < 0.05, Figure 4b). Anti-TREK-2 antibodies detected a band of ~60 kDa in HT-22 cells treated with LiCl, carbamazepine, paroxetine, and fluoxetine (Figure 4a). LiCl and carbamazepine did not affect TREK-2 expression levels. Paroxetine and fluoxetine slightly increased TREK-2 expression (paroxetine intensity: 1.6 ± 0.4 and fluoxetine intensity: 1.3 ± 0.2, Figure 4b). 

To assess the effect of antidepressants and mood stabilizers on whole-cell currents in HT-22 cells, we recorded whole-cell currents under same condition as recorded in TREK transfected cells before and after treatment with fluoxetine or LiCl. Fluoxetine inhibited the whole-cell currents, and LiCl increased the currents, like they did human TREK-1 currents (*n* = 13, Figure 4c,d). 

## 3. Discussion

Until now, TREK-1 has been viewed as a novel antidepressant target, because TREK-1 activity is inhibited by antidepressants and the deletion of TREK-1 (*KCNK2^−/−^*) resulted in antidepressant-like behavior in models of depression [3,5]. However, the precise role and antidepressant mechanism of *KCNK2^−/−^* is still an outstanding question. Our findings that inhibition of TREK-1 by antidepressants, activation by mood stabilizers, and modulation of TREK-1 expression extend the possibility that TREK-1 could be a potential target for treatment of not only depression but also bipolar disorder, while TREK-2 is likely to be involved in the regulation of major depression, since TREK-2 is modulated by only antidepressants and antipsychotics, but not mood stabilizers.

### 3.1. TREK-1 Channel as a New Target for Mood Disorder

Currently available drugs for the treatment/prophylaxis of bipolar disorder consist of mood stabilizers, such as LiCl, carbamazepine, valproate, lamotrigine, and atypical antipsychotics [9]. Of mood stabilizers, LiCl is the most commonly used drug for the treatment of bipolar disorder. More recently, anticonvulsant mood stabilizers have been established as an important addition to the classical mood stabilizer LiCl in the treatment of bipolar disorder. Anticonvulsants have been reported to have a variety of effects on bipolar disorder [10,11,12]. The efficacy of valproate and carbamazepine has been established for mania, and they may be more effective than LiCl in rapid cycling and dysphoric mania [13]. In this study, TREK-1 was seen to be more sensitive to anticonvulsants, particularly carbamazepine, than to the classical mood stabilizer LiCl. The establishment of valproate and carbamazepine as mood stabilizers stimulated the investigation and development of a new generation of anticonvulsants as potential mood stabilizers (e.g., lamotrigine). However, lamotrigine is less beneficial in mania [8], although it has efficacy in bipolar depression [14,15] and rapid cycling [16]. Our results also showed that lamotrigine had little or minimal effect on TREK-1 and TREK-2. Lamotrigine has been successful in mixed bipolar states in people who have not received adequate relief from LiCl, carbamazepine, and valproate. Here, TREK-1 channels were activated by LiCl, carbamazepine, and valproate. However, from this study, it appears that we can conclude is that TREK-1 channels do not serve as a target for lamotrigine. 

Interestingly, anti-TREK-1 antibodies detected two bands in HT-22 cells. In particular, the two TREK-1 bands were differentially modulated in response to paroxetine and fluoxetine: the upper band was downregulated or unchanged, whereas the lower band was significantly upregulated (*p* < 0.05). In contrast, LiCl and carbamazepine showed small increases in both bands, compared to untreated controls. These results give rise to a novel insight that antidepressants and mood stabilizers may modulate TREK-1 expression, although the mechanism of action is unknown at present. We cautiously suggest that the two bands might be approximated as a full-length TREK-1 (about 47 kDa) and a truncated TREK-1 (TREK-1_Δxx_, isoform lacking some residues of the intracellular N or C terminus). A recent study has reported that full-length TREK-1 and TREK-1_Δ1–56_ (isoform lacking 56 residues of the intracellular N terminus) are differentially expressed in a regional and developmental manner within the rat central nervous system [17]. Because HT-22 cells are derived from mouse brain, we speculate that this similar event might have also arisen in HT-22 cells. Moreover, our results are of interest because antidepressants are reported to induce a switch in TREK-1 isoforms. Expression of TREK-1_Δ1–56_ produced smaller outward currents and membrane depolarization than full-length TREK-1, appearing to act like a dominant negative form of TREK-1 [17]. A decrease in full-length TREK-1 and an increase in TREK-1_Δxx_ by antidepressants might mimic the knock-down effect of functional TREK-1 by siRNA. However, HEK-293 cells transfected with human TREK-1 did not show two bands in our experiment. Further study is needed to identify this difference between mouse and human TREK-1. In bipolar disorder, antidepressants might elevate mood from levels seen in depression through modulation of TREK-1, with continuous effects of antidepressants resulting in mania. Mania could also be controlled by activation of TREK-1 by mood stabilizers. Mood stabilizers are also used for the treatment of depressive patients in the clinical setting. In particular, mood stabilizers could potentially be used in combination with antidepressants when the introduction of various forms of treatment such as selective serotonin reuptake inhibitor (SSRI), serotonin and norepinephrine reuptake inhibitor (SNRI), norepinephrine and dopamine reuptake inhibitor (NDRI), norepinephrine and specific serotonin antagonist (NaSSa), and combinations of drugs with other mechanisms (e.g., SSRI + NDRI, SSRI + SNRI) are ineffective to patients. The concentrations of drugs modulating TREK channels in this study are higher than concentrations in blood. However, the dose can be different among organs and on symptoms. The concentration of fluoxetine in the brain is 10-fold higher than that in blood [18]. Although high concentrations of antidepressants and mood stabilizers work on TREK channels in vitro, the TREK-1 channel could not be excluded from an effective target for mood disorders, including bipolar disorder and depression, in vivo. 

### 3.2. Mood Stabilizers Can Discriminate TREK-1 from TREK-2, In Vitro

We have shown that both TREK-1 and TREK-2 currents are inhibited by several antidepressants and antipsychotics with IC_50_ values of ~40 μM, in agreement with earlier studies [2,3,19]. However, mood stabilizers showed differential effects on TREK-1 and TREK-2. TREK-2 was not activated by several mood stabilizers at drug concentrations that potently activated TREK-1 channels. TREK-1 and TREK-2 belong to the same two-pore domain K^+^ channel family and they share many properties. Both TREK-1 and TREK-2 channels are activated by free fatty acids, negative pressure, volatile anesthetics, acids, and heat; whereas they are inhibited by G-protein (G_s_ and G_q/11_) coupled receptor agonists [7]. These properties indicate that both TREK-1 and TREK-2 not only regulate cell excitability but may also become important during conditions of metabolic stress, when intracellular levels of free fatty acids and protons, cell volume, and body temperature are increased [20,21,22,23,24,25]. TREK-1 and TREK-2 are similarly modulated in general by stress. In addition, they have similar biophysical and pharmacological properties, with the exception of single-channel conductance at positive membrane potential. Therefore, it has been very difficult to isolate TREK-1 or TREK-2 from TREKs expressed in native primary cells. In addition to the implications for treatment of mood disorders, we propose that these mood stabilizers tested here can also be applied, in vitro, as a tool to distinguish TREK-1 from TREK-2 channels with application of intracellular low pH and ruthenium red [26,27,28].

## 4. Materials and Methods 

### 4.1. Cell Culture and Transfection of HEK-293 Cells

HEK-293 cells were seeded at a density of 2 × 10^5^ cells per 35 mm dish 24 h prior to transfection in Dulbecco’s modified Eagle’s medium (DMEM) containing 10% fetal bovine serum (FBS; Invitrogen, Grand Island, NY, USA) and 50 U/mL penicillin and streptomycin (Invitrogen). HEK-293 cells were co-transfected with human TREK-1 DNA (GenBank accession No. BC069462) or human TREK-2 DNA (GenBank accession No. AF385400) in pcDNA3.1 and pcDNA3.1/green fluorescent protein (GFP) using LipofectAMINE (Invitrogen) and Opti-MEM^®^ I Reduced Serum Medium (Invitrogen). For electrophysiological experiments, transfected cells were plated and grown on 12-mm microscope cover glasses, which were coated with poly-l-lysine for optimal cell attachment, in 35-mm culture dishes and maintained for 48 h at 37 °C in a humidified atmosphere of 95% air and 5% CO_2_. The cells expressing GFP were detected by epifluorescence with a microscope (Axiovert 135; Carl Zeiss Jena GmbH, Jena, Germany) equipped with a mercury lamp light source. Cells were used one to three days after transfection. All experiments were performed with the approval of the Research Ethics Committee of Gyeongsang National University.

### 4.2. Electrophysiological Studies

Electrophysiological recording was performed using a patch clamp amplifier (Axopatch 200, Axon Instruments, Union City, CA, USA). Whole-cell currents were recorded after canceling the capacitive transients. Single-channel and whole-cell currents were analyzed with the pCLAMP program (Version 9, Axon). Pipette and bath solutions contained (mM): 150 KCl, 1 MgCl_2_, 5 ethylene glycol tetraacetic acid (EGTA), and 10 hydroxyethyl piperazineethanesulfonic acid (HEPES ) (pH 7.3), and bath solution for recording of whole-cell current contained (mM): 135 NaCl, 5 KCl, 1 CaCl_2_, 1 MgCl_2_, 5 glucose, and 10 HEPES (pH 7.3). The pH was adjusted to desired values with HCl or KOH. To obtain IC_50_ and half maximal effective concentration (EC_50_) values for dose-dependent inhibition and activation, respectively, data were averaged and then fit with a standard sigmoid function. 

### 4.3. Western Blot Analysis

HT-22 neuronal cells were cultured in DMEM plus 10% FBS supplemented with 2 mM l-glutamate, 0.24% HEPES, 0.375% sodium bicarbonate, 100 U/mL penicillin, and 100 μg/mL streptomycin at 37 °C. Cells were treated for 24 h with either fluoxetine (30 μM), paroxetine (30 μ M), LiCl (1 mM), or carbamazepine (100 μM) to identify changes in TREK protein expression. Following treatment, HT-22 cells were harvested, washed three times with cold phosphate buffered saline (PBS), lysed in lysis buffer (RIPA buffer, Cell signaling technology, Danvers, MA, USA; 20 mM Tris-HCl (pH 7.5), 150 mM NaCl/L mM Na_2_EDTA, 1 mM EGTA, 1% NP-40, 1% sodium deoxycholate, 2.5 mM sodium pyrophosphate, 1 mM β-glycerophosphate, 1 mM Na_3_VO_4_, and 1 μg/mL leupeptin), incubated at 4 °C for 30 min, and centrifuged at 13,000 rpm (16,609× *g*, Micro 17TR, Hanil, Korea) for 30 min (at 4°C). After centrifugation, the supernatants were collected and quantified by the Bradford protein assay (Bio-Rad, Hercules, CA, USA) as total protein. The total protein samples (25 μg/lane) was separated on a 10% SDS-polyacrylamide gel and transferred to a polyvinylidene fluoride (PVDF) membrane (0.45 μm, Millipore, Bedford, MA, USA) in a buffer solution (Tris buffered saline (TBS); 25 mM Tris-base, 190 mM glycine, and 20% methanol) using a semi-dry blotter (Bio-Rad). After blocking with 5% fat-free milk and 0.05% Tween 20 in TBS for 1 h, the membranes were immunoblotted with anti-TREK-1 (Alomone Labs, Jerusalem, Israel/Chemicon, Temecula, CA, USA) and anti-TREK-2 (Alomone Labs) polyclonal antibodies, at 1:1000 dilutions at 4 °C overnight. After binding of horseradish peroxidase (HRP)-conjugated goat anti-rabbit antibody (1:3000; assay designs, Ann Arbor, MI, USA) at room temperature for 1 h, antigens were detected by enhanced chemiluminescence (ECL Plus kit; ELPIS, Taejeon, Korea) according to the manufacturer’s instructions.

### 4.4. Chemicals 

For the electrophysiological study, application of pharmacological agents including fluoxetine, paroxetine, citalopram, chlorpromazine, lamotrigine, LiCl, gabapentin, valproate, and carbamazepine in different concentrations always started with the lowest concentration. Stock solutions of fluoxetine (10 mM), citalopram (10 mM), chlorpromazine (100 mM), gabapentin (100 mM), valproate (1000 mM), and LiCl (1000 mM) were prepared in distilled water; carbamazepine (100 mM) in ethanol; and lamotrigine (100 mM) and paroxetine (20 mM) in dimethyl sulfoxide (DMSO), respectively, then diluted in bath solution to a working concentration. When ethanol or DMSO was used as a solvent, a solution containing an equivalent concentration was used as a control. Final ethanol and DMSO concentrations were 0.1% for carbamazepine, lamotrigine, and paroxetine. Unless otherwise stated, all chemicals were purchased from Sigma Chemical Co. (St. Louis, MO, USA). 

### 4.5. Data Analysis and Statistics 

LAS-4000 (Fujifilm corp., Tokyo, Japan), a luminescent image analyzer, was used to capture western blot images. Bands obtained from the blot were quantified by Sigma Gel image analysis software (version 1.0, Jandel Scientific, CA, USA) or Quantity One software (version 4.6.3) linked to a GS-800 calibrated densitometer (Bio-Rad). Relative protein levels were calculated by normalizing to α-tubulin concentrations. Student’s *t*-test was used, with *p* < 0.05 as the criterion for significance. Data are represented as mean ± SD unless specified.

## 5. Conclusions

In this study, we demonstrate for the first time that mood stabilizers modulate TREK-1, but not TREK-2. Our study suggests that TREK-1 may be a target for the treatment of bipolar disorder, which comprises various symptoms, such as psychiatric disturbance, depression, and mania together. Although we do not know the exact mechanisms of action by which TREKs are affected by both mood stabilizers and antidepressants, we can assume that a direct mechanism of activation will predominate over an indirect mechanism through signal transduction pathways since TREKs were found to be modulated under excised patch configuration. Further study will be needed to investigate the precise role of TREK channels in mood disorders, in animal models, and to elucidate the pharmacological mechanisms of activation in greater detail. Highlighting the potential role of TREK channels in mood disorders, a recent study reported that depression could be a consequence of the excitatory processes of mania and suggested a revision in the treatment of depressive illness [29]. TREKs are likely to contribute to the regulation of cell excitability directly or indirectly in bipolar disorders and represent an important target for research into the pathophysiology of mood disorders and the development of novel treatments for these diseases. 

## Abbreviations

5-HT	5-Hydroxytryptamine
LiCl	Lithium chloride
TREK	TWIK-related K^+^
K_2P_	Two-pore domain K^+^ channel
